# Pure Port Wine

**Published:** 1872-03

**Authors:** 


### PURE PORT WINE,

Of foreign manufacture, is perhaps not attainable in this country, except in the few families who have had it in their cellars for twenty or thirty years, saving it for medicinal or sacramental purposes. Yet "port wine" is to be had at every fourth-rate dram-shop, known, however, by all chemists, to have been made mainly out of water, logwood, sugar, and alcohol, without one drop of the juice of the grape. If port wine is to be used at all, it is well to know that it must be made from the juice of the grape alone, having thus more substance, more nutriment, than any of the European brands; as by the analysis of the department of Agriculture, at Washington City, it contains eighteen per cent. of the nutritive principle of the grape. As pure port wine will continue to be prescribed for years to come by educated physicians for certain derangements of the bowels and debilitated conditions of the system, and is of undoubted value in these directions, although a better result could be obtained in other ways, and as logwood wine would prove a positive injury, it is well to find out where an honest article can be had.

It is painful to remember how many good and learned men have indirectly encouraged the use of wine, on the plea that it prevented drunkenness by its keeping alcoholic liquors out of use, citing as proof, that they had travelled through France and other European countries where water was displaced by the use of wine, and that they never saw a single drunken man. The beer-drinking of England was also cited with the same views.

Everybody ought to know that the shops don't sell a drop of wine that does not contain alcohol; it is the alcohol in anything which makes a man drunk. It is with the express purpose of obtaining the effects of alcohol that wine and beer are used; and if they did not contain alcohol, they could not be sold at a dime a gallon to any regular drinker. If a man takes a bunch of grapes and presses the liquid portion into a goblet and drinks it, he is drinking the pure juice of the grape, but is not drinking " wine " in the common acceptation of the term. Wine really is the juice of the grape with alcohol in it; which alcohol gets into this juice of the grape by letting it stand a little while in the ordinary temperature of the vintage time of year.

You press out an apple, it is the juice of the apple; if you let it stand in apple-gathering time for several days, it has become cider, by alcohol having got into it. The longer the juice of the apple of our orchards stands, the more alcohol is found therein, by the natural laws of fermentation; and as wines, beers, and ciders are artificial stimulants, the more they are drank, the more frequently and the more largely the system requires them, yearns for them; in process of time they do not yield stimulus enough, and articles are used in their stead which contain a larger amount of alcohol; so that the habitual use of wine, and cider, and beer, leads uniformly and inevitably, sooner or later, to habitual drunkenness.

The indisputable facts of history show that for years before the rule of the Paris Commune in 1871, which was a rule of drunken debauchery, and murder, and assassination, wine had long since ceased to give the desired stimulus, and the use of absinthe was rapidly becoming apparently universal, worse than the most fiery brandy on the body, the brain, and the intellect; and at this very hour drunkenness in Switzerland is ten times more prevalent than it was a very few years ago; so that all the talk about the use of wine tending to prevent drunken habits is a most palpable fallacy. But if people will use wine in sickness, let that use be absolutely and always laid aside as soon as well enough to take a ride or walk. But for sacramental and medicinal purposes, Sylvester's Port Wine, at Lyons, New York, is the pure juice of the grape, with only the amount of alcohol which is generated by its own fermentation, as in cider.

